# EBV miRNA expression profiles in different infection stages: A prospective cohort study

**DOI:** 10.1371/journal.pone.0212027

**Published:** 2019-02-13

**Authors:** Anita Hartung, Oliwia Makarewicz, Renate Egerer, Matthias Karrasch, Anne Klink, Andreas Sauerbrei, Karim Kentouche, Mathias W. Pletz

**Affiliations:** 1 Institute of Infectious Diseases and Infection Control, Jena University Hospital, Jena, Germany; 2 Institute of Medical Microbiology, Jena University Hospital, Jena, Germany; 3 Department of Haematology and Medical Oncology, Jena University Hospital, Jena, Germany; 4 Institute of Virology and Antiviral Therapy, Jena University Hospital, Jena, Germany; 5 Clinic for Children and Youth Medicine, Jena University Hospital, Jena, Germany; University of North Carolina at Chapel Hill, UNITED STATES

## Abstract

The Epstein-Barr virus (EBV) produces different microRNAs (miRNA) with distinct regulatory functions within the infectious cycle. These viral miRNAs regulate the expression of viral and host genes and have been discussed as potential diagnostic markers or even therapeutic targets, provided that the expression profile can be unambiguously correlated to a specific stage of infection or a specific EBV-induced disorder. In this context, miRNA profiling becomes more important since the roles of these miRNAs in the pathogenesis of infections and malignancies are not fully understood. Studies of EBV miRNA expression profiles are sparse and have mainly focused on associated malignancies. This study is the first to examine the miRNA profiles of EBV reactivation and to use a correction step with seronegative patients as a reference. Between 2012 and 2017, we examined the expression profiles of 11 selected EBV miRNAs in 129 whole blood samples from primary infection, reactivation, healthy carriers and EBV seronegative patients. Three of the miRNAs could not be detected in any sample. Other miRNAs showed significantly higher expression levels and prevalence during primary infection than in other stages; miR-BHRF1-1 was the most abundant. The expression profiles from reactivation differed slightly but not significantly from those of healthy carriers, but a specific marker miRNA for each stage could not be identified within the selected EBV miRNA targets.

## Introduction

The Epstein-Barr virus (EBV) infects over 90% of adults and is one of the most frequent human viruses worldwide [1]. After infection, EBV enters B cells, where it persists throughout the host’s lifetime [2, 3]. Primary infections, especially in children, are often asymptomatic or are associated with minor flu-like symptoms [1, 2]. However, in industrialized countries, the timing of primary infection has shifted towards adolescence, and approximately 75% of these cases can be associated with infectious mononucleosis (IM) [4].

When infected with EBV, the virus particles penetrate into the pharynx and infect naïve B cells of the lymphatic tissue. There, however, no new virus is initially formed because the expression profile is reduced to 9 latency proteins. This stage is called latency III [5]. This expression pattern causes the naïve B cells to change into lymphoblasts and migrate to the germinal centre of the lymphoid tissue [6]. Here, a reduction of viral gene expression takes place, and the virus enters the latency II phase. After a further reduction of gene expression to produce only small regulatory RNAs (EBERs) [5], true latency (latency 0) is achieved, and the activated B cells change to resting memory B cells (MemB) cells, which then circulate in the blood. The proliferation of circulating MemB cells leads to the formation of latency phase I, which is characterized by the expression of the Epstein-Barr core antigen EBNA1 [7]. This protein ensures maintenance of the viral genome during division. EBNA1 is also the only viral protein that is produced in all phases of infection to protect the EBV genome as well as to inhibit spontaneous viral reactivation, thereby escaping the immune response [8]. Some of the circulating MemB cells migrate back into the lymphoid tissue of the oropharynx, where they differentiate into plasma cells. This leads to reactivation of the virus and entry into the lytic cycle. New virus particles are released within the lymphoid tissue and can infect other B cells or can be transmitted to other individuals [6, 9].

The virus can reactivate the lytic cycle during immunosuppression, stress, pregnancy or other infections [10, 11]. EBV causes a wide spectrum of diseases ranging from asymptomatic courses to rare diseases, including life-threatening haemophagocytic lymphohistiocytosis (HLH) or post-transplant lymphoproliferative disorder (PTLD) [2, 12]. In these conditions, the expression profiles of viral antigens are different, but EBV still maintains its latency (type I, II and III) [13, 14]. The incidence of severe chronic EBV infections or malignancies differs tremendously in different areas of the world. This difference has been linked to either increased genetically determined susceptibility to EBV infections, such as HLH in Asian or Native American children [15], or co-infections, such as malaria, which is a suspected cause for the increased incidence of Burkitt lymphoma in African children [16]. In contrast to other herpes viruses, no specific antiviral therapeutic options are available for EBV.

In 2004, EBV was the first virus shown to express small non-coding microRNAs (miRNAs) [17]. MiRNAs regulate post-transcriptional gene expression by base pairing to the 3’ untranslated region of mRNAs to block translation and/or cause mRNA cleavage [18], thereby influencing a variety of host cellular processes, such as apoptosis, proliferation or lipid metabolism [19]. EBV miRNAs are encoded in only 2 transcripts: BHRF1 and BART [17]. The BHRF1 transcript harbours 3 miRNAs, which are mainly expressed during the lytic cycle but are also expressed in latency stage III when the Cp and Wp promotors are still active [20]. In cell culture experiments, these miRNAs were identified as essential for the differentiation of naïve B cells into memory B cells [21]. The 22 BART miRNAs are highly expressed in all types of EBV latency [13] and are responsible for repressing lytic replication and maintaining latency [22].

Viral miRNAs control the metabolic stage of the cell by regulating the expression of viral and host genes. Therefore, they have been discussed as a potential diagnostic marker or as a therapeutic target. EBV miRNA expression profiles have been intensively studied in EBV-associated malignancies [23–26]. A few studies have investigated miRNA expression in primary infections [14, 27, 28], but nothing is known about miRNA expression profiles during reactivation.

In this study, the miRNA expression profiles in whole blood during EBV reactivation, primary infection and persistence (healthy carriers) of 11 EBV miRNAs were determined for the first time in a Caucasian cohort. The 11 targets were selected based on a previous publication investigating differences in the expression of certain EBV miRNAs during IM [29] (miR-BHFR1-1, miR-BHFR1-2, miBART-1-5p, miR-BART2-5p, miR-BART5-5p, miR-BART7-3p, miR-BART13-3p, miBART15-3p, miR20-5p). Additionally, miR-BART6-5p, which has been shown to maintain latency [30], and miR-BART9-3p, which is related to tumourigenesis and cell migration, were included [31]. The aim of this study was to identify promising miRNAs for targeted diagnostic or therapy of EBV-caused non-malignant disorders.

## Materials and methods

### Study population

In this observational study, patients with clinically suspected and laboratory-confirmed EBV infection and control patients hospitalized for non-EBV-associated reasons (e.g., surgery) were prospectively enrolled at the University Hospital Jena between September 2012 and July 2017. Patients with EBV-induced malignancies were excluded. EBV infection status was defined by routine diagnostics, including serological tests for EBV-specific antibodies and quantification of EBV DNA (viral load) using the artus EBV QS-RGQ PCR Kit (Qiagen, Hilden, Germany). A viral load >4 x 10^5^ in whole blood with detectable viral capsid antigen (VCA)-specific IgM and/or IgG but an absence of anti-EBNA-1-IgG was defined as ‘primary infection’ (PI). Positive anti-EBNA-1-IgG and anti-VCA-IgG titres with viral loads >10^3^ were defined as ‘reactivation’ (RA), whereas healthy carriers (HC) were positive for anti-EBNA-1-IgG and anti-VCA-IgG but negative for anti-VCA-IgM and anti-EA-IgM and without detectable viral load. The group of ‘seronegative’ (SN) subjects had no detectable viral load or EBV-specific antibodies [32] (Table 1).

**Table 1:** Comparative characteristics of the patients.

	PI group	RA group	HC group	SN group	P-value
Male	19 (54%)	31 (70%)	24 (67%)	11 (79%)	
Female	16 (46%)	13 (30%)	12 (33%)	3 (21%)	
Median age in years	13	37	39.5	1	/
Mean age in years ± SD	12.7 ± 9.3	35.9 ± 22.6	36.7 ± 20.3	4 ± 7.7	<0.0001
Mean Virus copies/mL ± SD	1 x 10^6^ ± 2.2 x 10^6^	6.1 x 10^4^ ± 1.3 x 10^5^	0	0	<0.0001
Origins of the collected samples:					
Paediatric Clinic	28	14	10	3	/
Transplantation Outpatient Department	-	17	19	-	/
Department of Hepatology/Infectiology	5	5	1	-	/
Paediatric Surgery	-	-	-	10	/
Department of Haematology/Oncology	2	3	1	-	/
Cardiovascular and Thoracic Surgery	-	2	11	-	/
Emergency Department	1	2	3	1	/
Other	1	1	1	-	/

PI, primary infection; RA, reactivation; HC, healthy carriers; SN, seronegative

In addition, two patients with histologically confirmed PTLD and one patient with HLH fulfilling the diagnostic criteria of McClain and Eckstein [33] were included, but they were analysed separately because EBV-induced lymphoproliferative disorders and malignancies were defined as exclusion parameters.

The study design was in accordance with the Helsinki Declaration, was approved by the ethic committee of the Jena University Hospital (no. 3530-08/12) and was registered in the German Clinical Trial Registry (DRKS00010713). Written informed consent was obtained from each patient or the parents of paediatric participants.

### Sample collection and RNA extraction

From adult participants, 9 mL, and from children between 1 and 6 years of age, 1.2 ml of peripheral blood was collected into the respective S-Monovette K-EDTA tubes (Sarstedt, Nümbrecht, Germany). From children up to 1 year of age, 500 μl capillary blood was taken using a K-EDTA Microvette (Sarstedt). The whole blood was aliquoted and stored at -80°C. The small RNA-enriched fraction was extracted from 300 μL of whole blood using the miRNeasy Mini Kit (Qiagen, Hilden, Germany) according to the manufacturer’s protocol. Qualitative analysis by an Agilent Bioanalyzer 2100 (Agilent Technologies, Santa Clara, USA) confirmed that primary miRNA (size < 50 bases) was present after preparation.

### Quantification of EBV miRNAs

A modified version of stem-loop RT-qPCR [34] was used for miRNA detection. Reverse transcription (RT) was performed as previously described [26] but with a final concentration of 50 nM for each RT primer. All RT reactions, including the controls (no reverse transcription (NRT) and no template control (NTC)), were performed in two independent replicates. The signals were enhanced by pre-amplifying 4 μL of the transcribed cDNA using the SsoAdvanced PreAmp Supermix (BioRad, Hercules, USA) and a 50 nM mixture of all specific forward and reverse primers (S1 Table) according to the manufacturer’s instructions. Subsequently, the pre-amplified samples (preAmp-DNA) were diluted to a ratio of 1:20 for EBV miRNA or 1:2000 for *hsa*-miR-16 PCR. Quantitative PCR (qPCR) was performed in 15-μL volumes, including 12.5 μL of SsoAdvanced Universal SYBR Green Supermix (BioRad), 1 μM each specific forward primer, 1 μM universal reverse primer [26] and 2 μL of diluted preAmp-DNA on a CFX Connect real-time PCR system (BioRad) as follows: 98°C for 30 s, followed by 40 cycles of 95°C for 15 s and 62°C for 30 s. Melting curve analysis was performed after each run. qPCR was performed in two independent replicates, and all samples were thus investigated 4 times.

The threshold was set at a fluorescence intensity of 200 relative fluorescence units (within the logarithmic Phase), and signals reaching the threshold within 36 cycles were considered for quantification.

### Calibration of RT-qPCR and mathematical models for quantification

Synthetic miRNAs of the EBV targets were purchased from Sigma-Aldrich (St. Louis, USA). A 1 pM mixture of all targets was prepared and further serially diluted 1:5 to obtain 2 x 10^5^ to 64 molecules per reaction. For calibration, RT and qPCR were performed as described above but without the pre-amplification step. Based on the calibration curve, the efficiency of qPCR for each miRNA species and the absolute miRNA copy numbers of the samples were calculated.

The relative expression ratio was calculated according to Eq (1) using the SN group as a control for the efficiency-corrected comparative ΔΔCt method as described by Pfaffl [35], where Ratio_ΔΔCt_ represents the relative expression ratio of target EBV miRNAs in the EBV-positive groups (sample) versus its averaged expression in the SN group (control); the expression levels were additionally normalized to human *hsa*-mir-16 as a reference [34, 36].

$$ratio\Delta \Delta Ct=\frac{{\left(E_{t\arg et}\right)}^{\Delta Ct_{t\arg et(control-sample)}}}{{\left(E_{reference}\right)}^{\Delta Ct_{reference(control-sample)}}}$$ (1)

Additionally, the miRNA expression levels were set in relation to the basal expression levels of the HC group.

### Statistical analysis

Statistical analyses were performed with GraphPad Prism version 5.00 for Windows (GraphPad Software). The Kruskal-Wallis test with Dunn’s multiple comparison post test was used for comparisons. Spearman correlation coefficient (r_s_) analysis was used to assess the relationship between viral load and EBV miRNA or paired expression of the miRNAs. Differences with p < 0.05 were considered statistically significant. Network graphics were drawn using Cytoscape version 3.6.1.

## Results

### Patient characteristics

Of the 157 collected blood samples, 129 samples were eligible for miRNA expression analysis. Eleven samples were excluded because they could not be assigned to any of the four pre-defined study groups, and 17 samples contained an insufficient volume of material (17.8% dropout). In total, 85 male and 44 female patients were enrolled. Males were predominant in the groups, except in the PI group, where gender was more balanced (Table 1). Most patients were enrolled in the Department of Paediatrics (42%), the Transplantation Outpatient Department (27.5%) and Department of Gastroenterology/Hepatology and Infectious Diseases (8.5%) (Table 1). The patients’ ages ranged from 4 months to 81 years with significant differences of the means (p < 0.001) between the groups (Table 1). As expected, the youngest patients (median age of 1 year) were found in the seronegative group, followed by the PI group (median age of 13 years). No significant difference in the age distribution was found between the RA and HC groups. The viral load in the RA group was significantly lower than in the PI group (6.1 x 10^4^ copies/mL vs 1 x 10^6^ copies/mL, p < 0.001). Viral loads were not detectable for HC and SN subjects since these groups are defined by this characteristic.

### Expression level of EBV miRNAs in the blood

**Fig 1:**
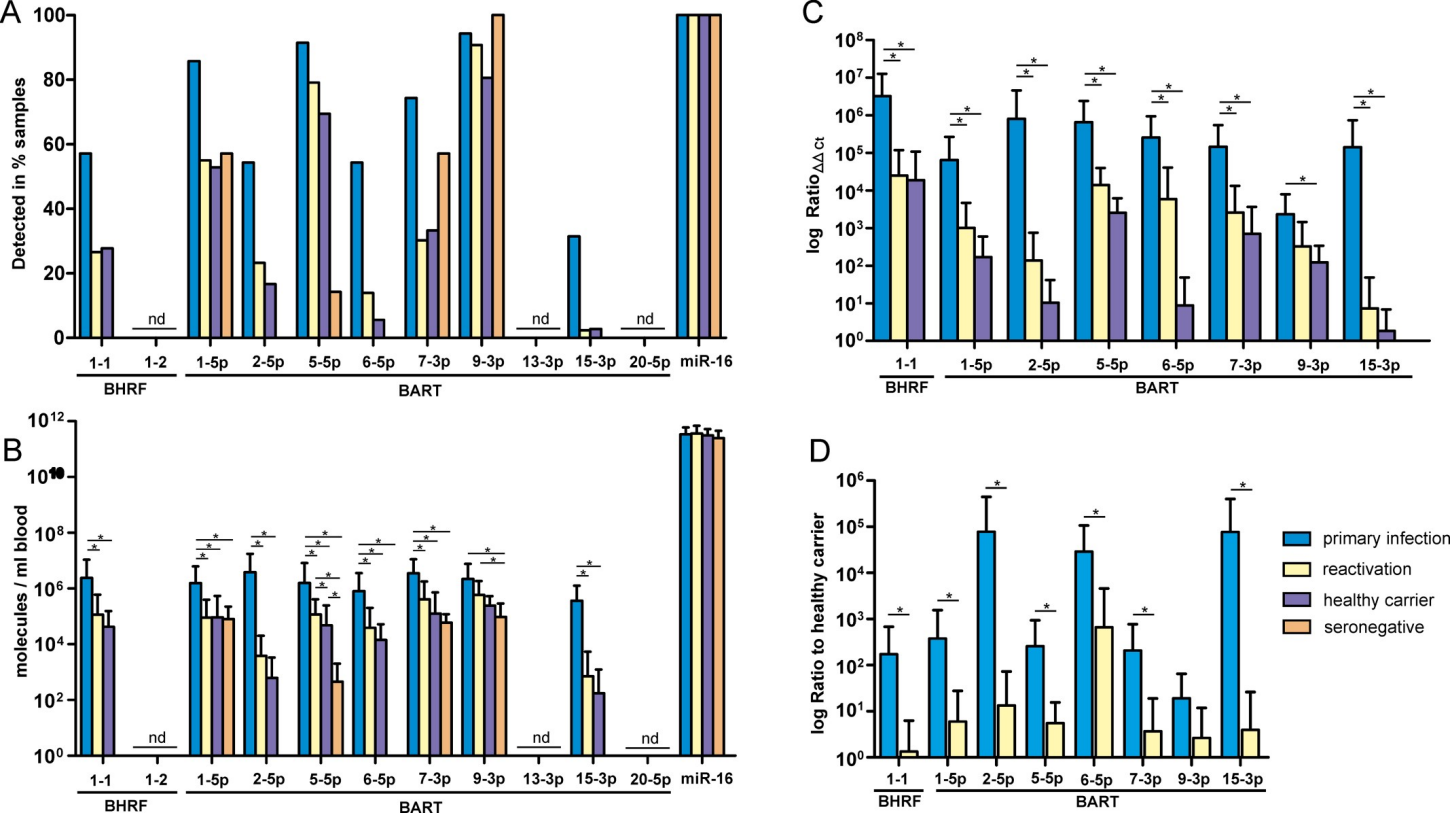
Comparison of EBV miRNA presence and respective expression levels during different stages of infection. (A) The number of samples with a positive miRNA PCR signal in the respective group is shown as a percentage. (B) Non-normalized averaged miRNA copy numbers for all 4 study groups. Calibration was performed for each miRNA species using synthetic miRNAs of identical sequence. (C) Relative expression levels were normalized to the SN group. Only detectable miRNAs are shown. Relative expression was calculated by the efficiency-corrected ΔΔCt method [35]. (D) Expression ratios in relation to the HC group, which represents the basal miRNA levels in EBV-positive patients. Expression levels are shown as the mean and standard deviation. Comparisons were performed using the Kruskal-Wallis test with Dunn’s multiple comparison test. Significance was expressed as asterisk (* indicates *p* < 0.05).

Absolute copy numbers and relative expression levels were assessed for 2 BHRF1 miRNAs and 9 well-investigated BART miRNAs. The endogenous reference *hsa*-mir-16 proved to be robust due to its stable expression in all groups (S1 Fig). MiR-BHRF1-2, miR-BART13-3p and miR-BART20-5p were not been detected in any of the samples and were therefore excluded from further analysis (Fig 1A).

The presence of the different miRNAs was more frequent in the PI group: miR-BART1-5p, miR-BART5-5p and miR-BART9-3p were found in 85–95% of the samples, and miR-BART7-3p was found in 75%; miR-BHRF1-1 and miR-BART6-5p were detected in approximately 55% of the samples, and miR-BART15-3p was found in only 31%. In the RA and HC groups, the presence of EBV miRNAs was noticeably lower (Fig 1A). Only miR-BART5-5p was found in more than 70% of samples in these groups, whereas only one positive sample in each group was found for miR-BART15-3p. miR-BART9-3p was present in all SN samples, whereas miR-BART1-5p and miR-BART7-3p were each present in 57% of the SN samples.

The absolute copy number of EBV miRNAs in whole blood was generally significantly higher (p < 0.05) in the PI group than in the other groups, except for miR-BART9-3p, which showed no significant difference between the PI and RA groups (Fig 1B). The signals obtained for the SN group were relatively high for miR-BART9-3p > miR-BART1-5p > miR-BART7-3p > miR-BART5-5p, indicating strong background noise due to unspecific amplification by the corresponding primers (mandatory normalization).

After normalization to the SN control group to reduce false-positive signals and consider qRT efficiency (Fig 1C), miR-BHRF-1 was the most abundant EBV miRNA in all groups, with similar expression levels in the RA and HC groups. The BART miRNAs showed group-specific expression levels. They were highest in the PI group and decreased gradually from the RA group to the HC group, with the strongest differences for miR-BART15-3p and miR-BART2-5p (5 log-magnitudes higher in PI compared with HC). The smallest difference was found for miR-BART9-3 (less than 2 log-magnitudes higher). A strong but not significant difference between the RA and the HC groups was observed for only miR-BART6-5p (> 2.5 log-magnitudes higher).

Comparing the differences in miRNA expression between the PI and RA groups additionally normalized to the HC group (Fig 1D), all but miR-BART9-3p showed significantly higher expression in the PI group. The strongest differences were observed for miR-BART15-3p and miR-BART2-5p (5 log-magnitudes higher than HC and approximately 4 lag magnitudes higher than RA).

### Correlation between the viral load and miRNA expression profile

**Fig 2:**
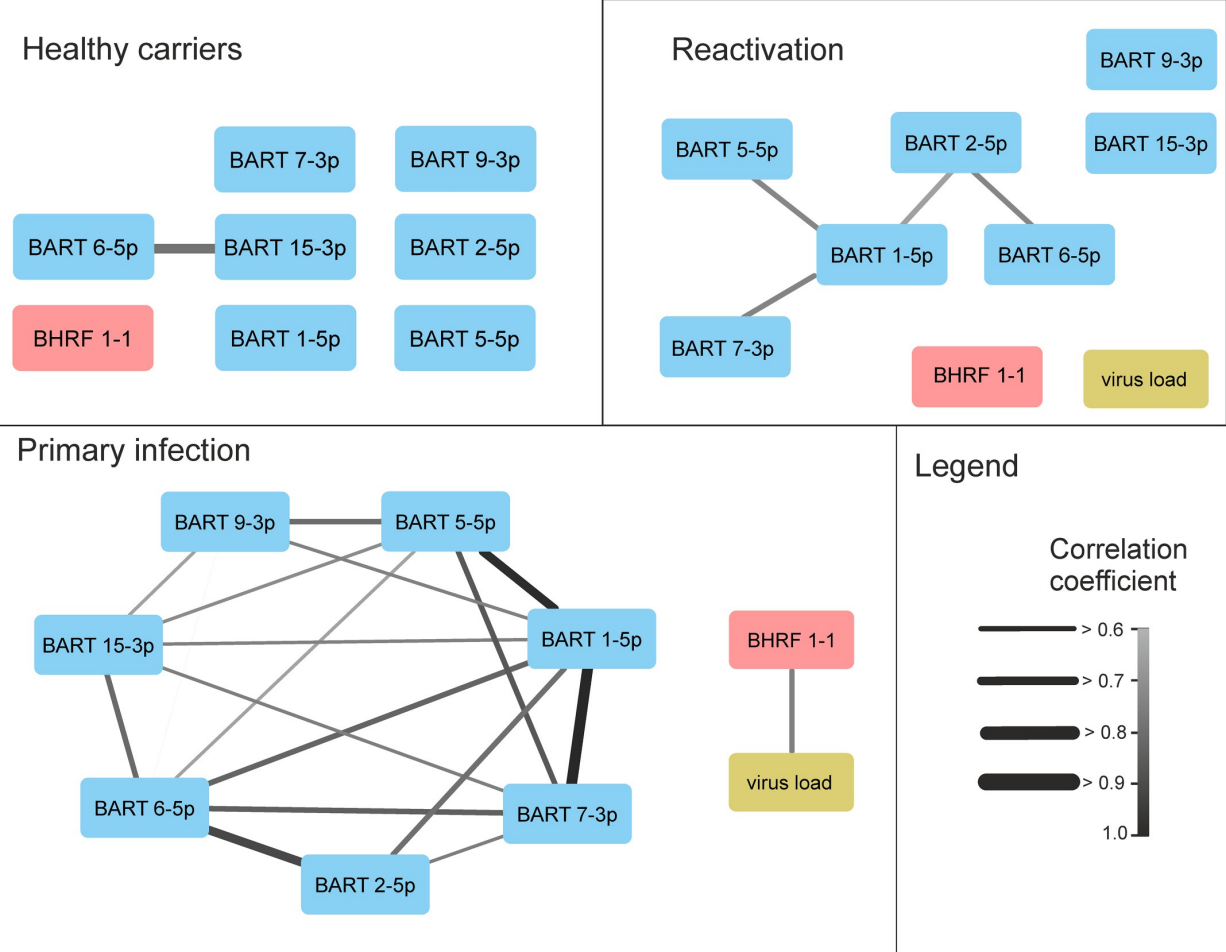
Pairwise correlations of the miRNAs and viral load in the different patient groups. The grey scale as well as the line sizes correspond to the respective Spearman correlation coefficient (rs).

The viral load was correlated most strongly with miR-BHRF1-1 and in the PI group (r_s_ = 0.67, p < 0.00001) (Fig 2 and S2 Table). No correlations (0.6 > r_s_ <-0.6) with other miRNAs were obtained, and no correlation of any miRNA with viral load was observed in the other groups.

### Pairwise correlations of miRNAs within infection states

The expression levels of the EBV miRNAs within the study groups were pairwise correlated. A positive correlation was assumed when the corresponding Spearman correlation factor (r_S_) was higher than 0.6; a negative correlation (r_S_ < -0.6) was not found for any miRNA pair (S2 Table).

In the PI group, miR-BART1-5p positively correlated with all BART miRNA species (Fig 2), and the highest correlations were with miR-BART7-3p (r_S_ = 0.902) and miR-BART5-5p (r_S_ = 0.890). MiR-BART2-5p highly correlated with miR-BART6-5p (r_S_ = 0.824, p < 0.001). In the RA group, the correlations between miRNA species were substantially lower (0.66 < r_S_ < 0.68) and were found for only four pairs: miR-BART1-5p with miR-BART2-5p, miR-BART5-5p or miR-BART7-3p and miR-BART2-5p with miR-BART6-5p. In the HC group, some correlation (r_S_ = 0.717) was observed only between miR-BART6-5p and miR-BART15-3p.

### EBV miRNA expression in HLH and PTLD

The miRNA profiles of a patient with HLH and two patients with PTLD, who were all excluded from the previous analysis and analysed separately, showed that miR-BHRF1-2, miR-BART13-3p and miR-BART20-5p and the less prevalent miR-BART6-5p and miR-BART15-3p were not detectable in these samples.

**Fig 3:**
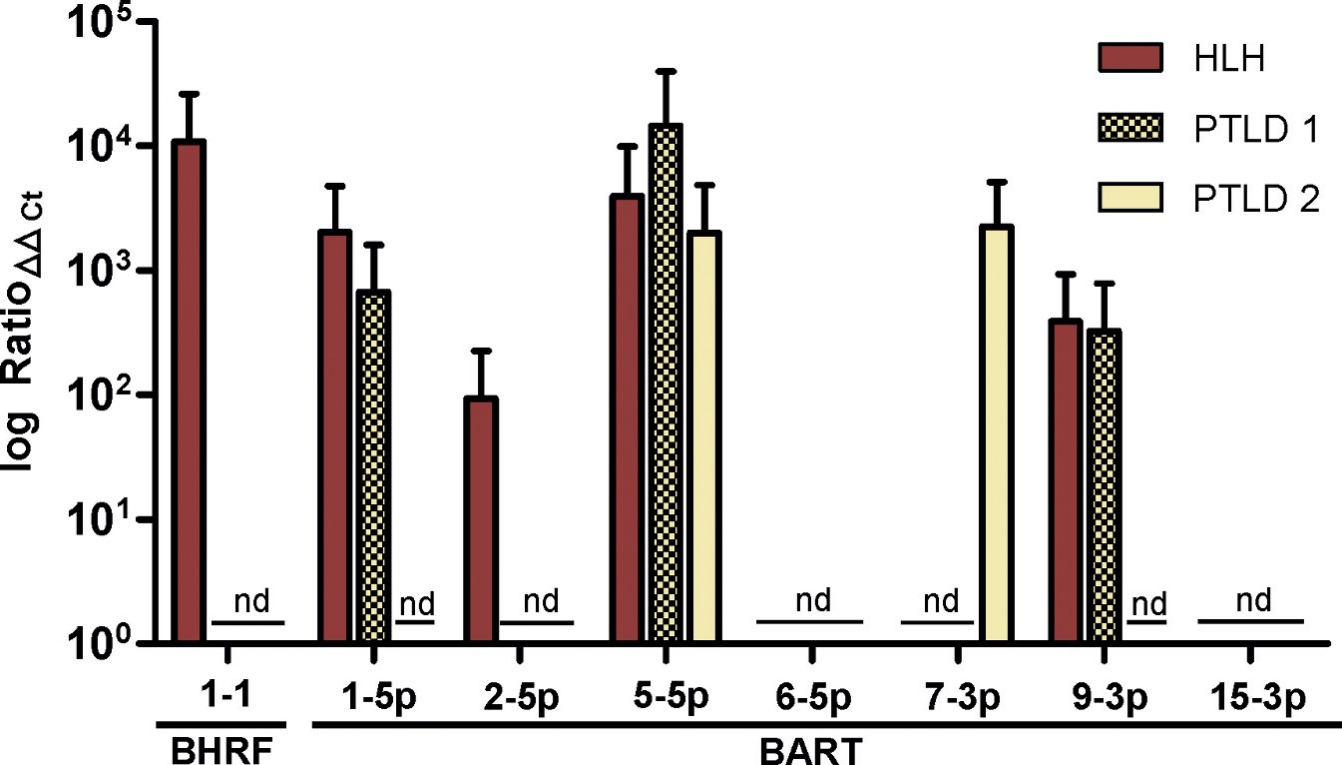
Comparison of the expression profiles of an HLH sample and two PTLD samples. Samples were collected from one patient with haemophagocytic lymphohistiocytosis (HLH) and two patients with post-transplant lymphoproliferative disorder (PTLD) and separately analysed. Expression levels were calculated by the efficiency-corrected ΔΔCt method.

In the HLH sample, miR-BART6-5p, miR-BART7-3p and miR-BART15-3p were absent; the expression levels of the other EBV miRNA species were similar to those in the RA group (Fig 3).

The two PTLD samples showed more variability in the expression profiles; while miR-BART6-5p and miR-BART15-3p were not expressed in either PTLD sample, miR-BART7-3p was expressed in one of the samples, and miR-BART9-3p and miR-BART1-5p were found in the other sample; the levels of all miRNAs were similar to those in the RA group. In contrast to the HLH sample, no expression of miR-BHRF1-1 and miR-BART2-5p was observed.

## Discussion

MiRNAs are recognized as future targets for therapeutics or even as therapeutics themselves [37], provided that the expression profile can be unambiguously correlated to a specific pathology or stage of disorder. Viral miRNA might provide new therapeutic options for many diseases so far unresponsive to therapy such as EBV-induced disorders. However, valid miRNA quantification has been difficult due to the short sequence length (∼22 nt in length), which increases the risk of primer overlap, self-annealing and auto-amplification [38]. Additionally, viral miRNA quantification is even more difficult due to high sequence identity to human RNA species, which often results in unspecific signals. These technical difficulties have resulted in a number of contradictory and sometimes confusing publications. For example, the EBV miR-BART7-3p was predominantly detected in studies–even in samples from seronegative volunteers–using common stem-loop RT-PCR [26, 28, 34, 38, 39]. This result was probably due to auto-amplification caused by self-annealing of the primers [38]. Some viral miRNAs might have also been falsely described as highly expressed, e.g., miR-BART13-3p [14, 27], by using poly(A)-tailing combined with SYBR green qPCR. Even when we applied a modified, more specific qPCR protocol, we obtained an unspecific PCR product for this miRNA that was confirmed by electrophoretic analysis (S2 Fig). Additionally, certain miRNAs, e.g., miR-BART9-3p, showed high expression levels but had similarly sized amplification products in seronegative samples, which strongly complicated the quantification. A possible explanation is that false-positive signals in the PCR occurred due to cross-reactivity of human miRNAs since EBV miRNAs exhibit high sequence similarities to mimic human miRNA species. Therefore, in this study, we optimized the detection protocol by i) adjusting the primer sequences to reduce unspecific signals caused by self-annealing, ii) including normalization against the SN group to subtract the unspecific background signals caused by human RNA species, and iii) calculating the relative expression levels by the efficiency-corrected ΔΔCt method [35] to account for different efficiencies for poorly expressed miRNAs. The primary aim of this study was to identify differentially expressed miRNA species in reactivation and primary infection; thus, we additionally normalized the profiles to basal levels in the HC group.

In our study, three of the 11 investigated EBV miRNAs, miR-BHRF1-2, miR-BART13-3p and miR-BART20-5p, were not expressed at all. All other EBV miRNAs showed generally significantly higher expression during PI than in other stages. It is surprising that no signal for miR-BHRF1-2 was found in the PI group because this miRNA (together with miR-BHRF1-3, miR-BART1, -2, -10-, and -22) cooperatively co-inhibits production of the IL-12-family cytokines responsible for the differentiation of CD4^+^ T-cells, which play a crucial role in the defence against the first stage of viral infections and are therefore a primary target of EBV immediately after B-cell infection [40]. On the other hand, expression of miR-BHRF1-2 was shown to be only visible within the first few hours of the infection [21] and therefore might be missed in patient samples.

MiR-BHRF1-1 was the most abundant EBV miRNA species in all groups and showed significantly higher expression in the PI group. This result is due to its central role in B-cell differentiation and the inhibition of apoptotic processes [21] and is in line with other studies [14, 27, 28, 41]. This miRNA could not be detected in 40% of PI samples but was observed in 25% of RA samples and 27% of HC samples, which is most likely due to its rapid decrease in expression after symptom onset as observed in previous studies [27]. Thus, miR-BHRF1-1 is unreliable for discriminating between different stages of infection. Like miR-BHRF1-1, miR-BART2-5p and miR-BART6-5p do not offer satisfactory discriminatory performance mainly because they are rarely expressed. MiR-BART1-5p, miR-BART7-3p and miR-BART9-3p showed a relatively high and frequent basal signal in SN patients, reducing their diagnostic accuracy and disqualifying these miRNA species as therapeutic targets. MiR-BART15-3p, which was sporadically identified at low levels in RA and HC samples, was highly expressed in PI samples. Thus, this miRNA might act as an additional positive predictor of PI in a test when combined with more reliable markers. Good results (high prevalence and significant differences) highlighted miR-BART5-5, which is known to inhibit apoptosis and maintain latency [42]. We found also a strong positive correlation among the expression of miR-BART5-5p, miR-BART1-5p and miR-BART7-3p, which also contribute to the inhibition of apoptosis and lytic replication [20, 21, 43], indicating co-regulation.

Separate analysis of the 2 PTLD samples showed different miRNA patterns, in line with a recent study that identified 2 different profiles of EBV miRNA expression for PTLD [44]. Interestingly, miR-BHRF1-1 and miR-BART2-5p were expressed in the HLH sample but not in the PTLD samples.

Additionally, we examined the paired expression of miRNAs and their correlation with viral load in whole blood. *Kawano* et al. reported positive correlations between 9 EBV miRNAs and EBV DNA copy number in whole blood [28]. In contrast to the previous study, only miR-BHRF1-1 was correlated with viral load.

In summary, reactivation could not clearly be differentiated from persistence (latency) by the miRNA profiles because the expression levels of the selected miRNA species in the RA and HC groups showed almost no significant differences. Moreover, none of the investigated miRNAs were able to discriminate between the pre-defined groups with sufficient reliability for diagnostic tests. One of the major problems for a valid analysis of the EBV-miRNA expression levels represents the seronegative cohort: On the one hand, it is essential to eliminate the background of cross-reactive human miRNA species; On the other hand, with a prevalence of more than 90% [1] and an high probability (60.6%) of EBV infection within the first 2 years of age [45] it remains very difficult to assemble an representative SN group that consists almost entirely of infants, from whom only limited blood sample volumes can be obtained limiting the number of testings or analysis. Thus, the current standard of viral load and serology seems to be superior for EBV diagnostics. In our opinion, the reliability of miRNA-based differentiation of EBV infections might improve if multiple miRNA species are combined in an array to increase their forecasting power. Additional and more suitable miRNA marker candidates might be identified by investigating additional EBV miRNA species. It must be noted that the generally low concentration of EBV miRNAs in whole blood might represent the greatest problem for valid miRNA-based diagnostic tests of EBV. The high sequence similarity with human miRNAs represents another problem. Therefore, due to the unsophisticated sensitivity and specificity, PCR-based methods for viral miRNA detection might be less suitable in general.

## Supporting information

S1 Table Primers used in this study. (DOCX)

S2 Table Pairwise correlations of EBV miRNA expression and the viral load. The Spearman’s correlation coefficients (rs) were pairwise determined. Red underlined cells (intensity increases with increasing rs) indicates a positive correlation that was assumed at rs > 0.6.
(DOCX)

S1 Fig Boxplot and statistics (below) of the Ct value distribution of the human normalizer miR-16. The medians are shown as horizontal lines within the boxes (25–75 percentiles) and the 5–95 percentiles as vertical lines. Statistical analysis by One-way ANOVA (Kruskal-Wallis test) with Dunn’s Multiple Comparison revealed any significant differences of the Ct values between the 4 study groups.
(DOCX)

S2 Fig Analysis of the amplification products of miR-BART13-3p primers. (A) The melt peak analysis after qPCR of miR-BART13-3p revealed a shift to lower temperature of the PCR-product for the sample (black) and the no reverse transcription control (NRT, blue) of 1–2°C compared to the positive control (orange) indicating a shorter PCR-product, which was confirmed by gel electrophoresis as unspecific (B).
(DOCX)

S1 File CT values. (XLSX)
