# Supplementary material for: EBV miRNA expression profiles in different infection stages: A prospective cohort study
Source: PLoS One. 2019 Feb 13;14(2):e0212027. doi: 10.1371/journal.pone.0212027 (PMC6373943; doi:10.1371/journal.pone.0212027)
Supplement: S1 Table — (DOCX) [file pone.0212027.s001.docx]

**Table S1. Primers used in this study.**

| Primer | Sequence 5’ – 3’ |
| --- | --- |
| BHRF1-1 RT | CTCAACTGGTGTCGTGGAGTCGGCAATTCAGTTGAGAACTCCGG |
| BHRF1-1 for | CGTAACCTGATCAGCC |
| BART1-5p RT | CTCAACTGGTGTCGTGGAGTCGGCAATTCAGTTGAGACAGCACG |
| BART1-5p for | TGTCTTAGTGGAAGTGACGT |
| BART2-5p RT | CTCAACTGGTGTCGTGGAGTCGGCAATTCAGTTGAGACAGCACG |
| BART2-5p for | TATTTCTGCATTCGCC |
| BART5-5p RT | CTCAACTGGTGTCGTGGAGTCGGCAATTCAGTTGAGCGATGGGC |
| BART5-5p for | CGTCAAGGTGAATATAGCT |
| BART6-5p RT | CTCAACTGGTGTCGTGGAGTCGGCAATTCAGTTGAGCCTATGGA |
| BART6-5p for | TAAGGTTGGTCCAATCC |
| BART7-3p RT | CTCAACTGGTGTCGTGGAGTCGGCAATTCAGTTGAGCCCTGGAC |
| BART7-3p for | TGCATCATAGTCCAGTGTC |
| BART9-3p RT | CTCAACTGGTGTCGTGGAGTCGGCAATTCAGTTGAGACTACGGGA |
| BART9-3p for | TCTGTAACACTTCATGGG |
| BART13-3p RT | CTCAACTGGTGTCGTGGAGTCGGCAATTCAGTTGAGTCAGCCGT |
| BART13-3p for | GTGTAACTTGCCAGGG |
| BART15-3p RT | CTCAACTGGTGTCGTGGAGTCGGCAATTCAGTTGAGTCAAGGAA |
| BART15-3p for | GCT GTCAGTGGTTTTGT |
| BART20-5p RT | CTCAACTGGTGTCGTGGAGTCGGCAATTCAGTTGAGGGAATGAA |
| BART20-5p for | AAGTAGCAGGCATGTC |
| miR16 RT | GTCGTATCCAGTGCAGGGTCCGAGGTATTCGCACTGGATACGACCGCCAA |
| miR16 for | CGCGCTAGCAGCACGTAAAT |
| miR16 rev | GTGCAGGGTCCGAGGT |
| EBV miRNA rev | GTGTCGTGGAGTCGGCA |
