# Supplementary material for: EBV miRNA expression profiles in different infection stages: A prospective cohort study
Source: PLoS One. 2019 Feb 13;14(2):e0212027. doi: 10.1371/journal.pone.0212027 (PMC6373943; doi:10.1371/journal.pone.0212027)
Supplement: S2 Table — The Spearman’s correlation coefficients (rs) were pairwise determined. Red underlined cells (intensity increases with increasing rs) indicates a positive correlation that was assumed at rs > 0.6. (DOCX) [file pone.0212027.s002.docx]

**S2 Table. Pairwise correlations of EBV miRNA expression.** The Spearman’s correlation coefficients (rs) were pairwise determined. Red underlined cells (intensity increases with increasing rs) indicates a positive correlation that was assumed at rs > 0.6.

|  |  | BHRF1-1 | BART1-5p | BART2-5p | BART5-5p | BART6-5p | BART7-3p | BART9-3p |
| --- | --- | --- | --- | --- | --- | --- | --- | --- |
| **Primary infection** | BART1-5p | 0.533 |  |  |  |  |  |  |
|  | BART2-5p | 0.398 | 0.726 |  |  |  |  |  |
|  | BART5-5p | 0.599 | 0.890 | 0.584 |  |  |  |  |
|  | BART6-5p | 0.372 | 0.752 | 0.824 | 0.600 |  |  |  |
|  | BART7-3p | 0.599 | 0.902 | 0.691 | 0.785 | 0.766 |  |  |
|  | BART9-3p | 0.232 | 0.684 | 0.463 | 0.734 | 0.495 | 0.554 |  |
|  | BART15-3p | 0.380 | 0.684 | 0.511 | 0.657 | 0.741 | 0.688 | 0.606 |
| **Reactivation** | BART1-5p | 0.400 |  |  |  |  |  |  |
|  | BART2-5p | 0.283 | 0.606 |  |  |  |  |  |
|  | BART5-5p | 0.160 | 0.668 | 0.326 |  |  |  |  |
|  | BART6-5p | 0.286 | 0.505 | 0.671 | 0.346 |  |  |  |
|  | BART7-3p | 0.426 | 0.675 | 0.538 | 0.414 | 0.595 |  |  |
|  | BART9-3p | -0.358 | -0.054 | -0.086 | 0.289 | 0.061 | -0.054 |  |
|  | BART15-3p | 0.324 | 0.260 | 0.302 | 0.250 | 0.392 | 0.229 | -0.037 |
| **healthy control gro** | BART1-5p | 0.580 |  |  |  |  |  |  |
|  | BART2-5p | 0.157 | 0.259 |  |  |  |  |  |
|  | BART5-5p | 0.055 | 0.322 | 0.066 |  |  |  |  |
|  | BART6-5p | -0.148 | 0.196 | 0.233 | 0.139 |  |  |  |
|  | BART7-3p | 0.438 | 0.382 | 0.246 | -0.212 | 0.169 |  |  |
|  | BART9-3p | -0.297 | -0.037 | -0.158 | 0.179 | -0.068 | -0.279 |  |
|  | BART15-3p | -0.103 | 0.112 | 0.363 | 0.041 | 0.717 | 0.339 | -0.269 |
