# Supplementary material for: EBV miRNA expression profiles in different infection stages: A prospective cohort study
Source: PLoS One. 2019 Feb 13;14(2):e0212027. doi: 10.1371/journal.pone.0212027 (PMC6373943; doi:10.1371/journal.pone.0212027)
Supplement: S1 Fig — The medians are shown as horizontal lines within the boxes (25–75 percentiles) and the 5–95 percentiles as vertical lines. Statistical analysis by One-way ANOVA (Kruskal-Wallis test) with Dunn’s Multiple Comparison revealed any significant differences of the Ct values between the 4 study groups. (DOCX) [file pone.0212027.s003.docx]

**
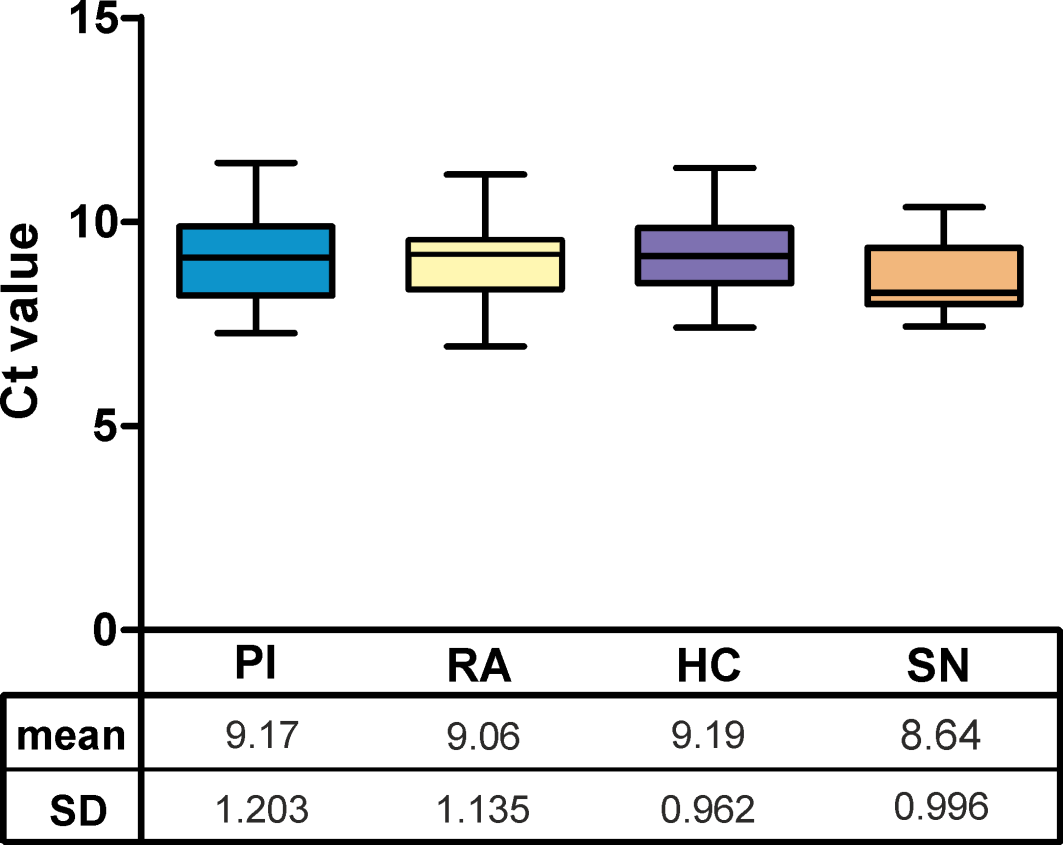
**

**S1 Fig. Boxplot and statistics (below) of the Ct value distribution of the human normalizer miR-16.** The medians are shown as horizontal lines within the boxes (25 – 75 percentiles) and the 5-95 percentiles as vertical lines. Statistical analysis by One-way ANOVA (Kruskal-Wallis test) with Dunn’s Multiple Comparison revealed any significant differences of the Ct values between the 4 study groups.
