# Supplementary material for: EBV miRNA expression profiles in different infection stages: A prospective cohort study
Source: PLoS One. 2019 Feb 13;14(2):e0212027. doi: 10.1371/journal.pone.0212027 (PMC6373943; doi:10.1371/journal.pone.0212027)
Supplement: S2 Fig — (A) The melt peak analysis after qPCR of miR-BART13-3p revealed a shift to lower temperature of the PCR-product for the sample (black) and the no reverse transcription control (NRT, blue) of 1–2°C compared to the positive control (orange) indicating a shorter PCR-product, which was confirmed by gel electrophoresis as unspecific (B). (DOCX) [file pone.0212027.s004.docx]

**
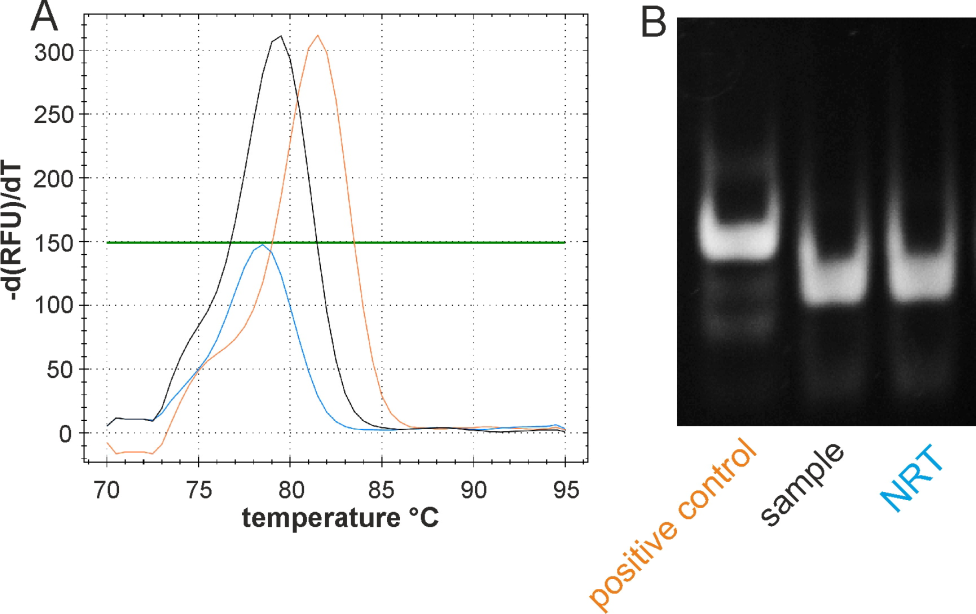
**

**S2 Fig. Analysis of the amplification products of miR-BART13-3p primers.** (A) The melt peak analysis after qPCR of miR-BART13-3p revealed a shift to lower temperature of the PCR-product for the sample (black) and the no reverse transcription control (NRT, blue) of 1-2 °C compared to the positive control (orange) indicating a shorter PCR-product, which was confirmed by gel electrophoresis as unspecific (B).
